# Transient reduction in macular deep capillary density on optical coherence tomography angiography after phacoemulsification surgery in diabetic patients

**DOI:** 10.1186/s12886-020-01605-8

**Published:** 2020-08-17

**Authors:** Zaowen Wang, Erqian Wang, Youxin Chen

**Affiliations:** 1 Department of Ophthalmology, Peking Union Medical College Hospital, Beijing, 100730 China; 2 Key Laboratory of Ocular Fundus Diseases, Chinese Academy of Medical Sciences, Beijing, 100730 China

**Keywords:** Phacoemulsification surgery, Macular edema, Optical coherence tomography angiography, Vessel density, Diabetes

## Abstract

**Background:**

To evaluate macular microvascular changes and associated factors in diabetic patients following uncomplicated phacoemulsification surgery.

**Methods:**

In this prospective observational study, we enrolled diabetic patients and non-diabetic controls who underwent phacoemulsification surgery. Participants were examined at postoperative day 1 (POD1), 10 (POD10), 30 (POD30), and 90 (POD90), using macular 3x3mm OCT angiography scan (RTVue-XR Avanti; Optovue, Inc., Fremont, CA). Integrated automated algorithms were used to quantify parafoveal vessel density (VD) in superficial capillary plexus (SCP) and deep capillary plexus (DCP). To minimize measurement bias, subjects with corneal edema or capsular opacity at any postoperative visit were excluded.

**Results:**

The study included 21 eyes of 21 diabetic patients and 21 eyes of 21 non-diabetic controls. In diabetic patients, no significant change in SCP-VD could be detected (*P* = 0.57); DCP-VD reduced from 50.24 ± 2.33% at POD1 to 48.33 ± 3.07% at POD30 (*P* = 0.019), and restored to 50.74 ± 3.44% at POD90 (*P* = 1.00). The DCP-VD change at POD30 in diabetic patients (− 1.90 ± 2.61%) was significantly different from that in controls (1.31 ± 2.61%) (*P* < 0.001). The amount of DCP-VD reduction was correlated with foveal and parafoveal thickening (*r* = 0.431, *P* = 0.051 and *r* = 0.514, *P* = 0.017, respectively), high cumulative dissipated energy (*P* = 0.032) and increased hemoglobin A1c concentration (*P* = 0.037).

**Conclusions:**

Phacoemulsification in diabetic patients caused transient reduction in DCP-VD, which was associated with poor glycemic control, surgical trauma, and postoperative macular thickening. Our results added a new dimension to our understanding of the complex biologic effects of cataract surgery in diabetic subjects.

## Background

Phacoemulsification cataract surgery is one of the most common anterior segment surgical procedures. Although it is now safer than before, complications are occasionally seen. Pseudophakic cystoid macular edema (PCME) is one of the important posterior segment complications with a baseline incidence rate of 1.17% [1]. Diabetes has been associated with increased incidence of PCME [1, 2], but how diabetes increases the risk remains unclear.

With the advent of optical coherence tomography angiography (OCTA), macular microvasculatures, including superficial and deep capillary plexus, can be visualized and quantified non-invasively [3, 4]. In previous studies which focused on non-diabetic subjects, cataract surgery was reported to cause an increase in macular microvasculature vessel density (VD) [5–7]. However, none of the previous studies included diabetic subjects, and how cataract surgery influence macular microvasculature networks in diabetic eyes remains unknown.

The current study focused on diabetic patients and aimed to study how their macular microvasculature networks responded to phacoemulsification surgery, compared with non-diabetic patients. We also aimed to investigate whether the microvascular changes were associated with postoperative macular thickening or baseline clinical characteristics.

## Methods

The study followed the tenets of the Declaration of Helsinki. Ethics committee of Peking Union Medical College Hospital approved the study protocol. All participants gave informed consent.

### Patients

Patients with type 2 diabetes undergoing phacoemulsification cataract surgery with an experienced cataract surgeon (Z.W.) between October 2018 and April 2019 were consecutively evaluated for this prospective study. We also recruited age- and lens grading-matched non-diabetic subjects as controls during the same period. Participants were excluded at baseline if they had: 1) age younger than 50 years or older than 80 years; 2) high myopia of over − 6.00 diopters or over 26.50 mm in axial length; 3) diabetic macular edema, intraretinal microcyst or any other diabetic maculopathy at baseline; 4) retinal diseases except for mild or moderate nonproliferative diabetic retinopathy (NPDR); 5) history of retinal laser or intravitreal injection; or 6) other systemic conditions including but not limited to uncontrolled hypertension, autoimmune diseases, and malignant tumor. Eligible subjects underwent phacoemulsification surgery and were followed up at postoperative day 1 (POD1), 10 (POD10), 30 (POD30), and 90 (POD90). Subjects were further excluded for final analysis if they: 1) developed intraoperative or postoperative complication except for cystoid macular edema; 2) exhibited postoperative corneal edema or posterior capsule opacity that might interfere with accurate OCTA measurement at any postoperative visit; 3) failed to generate OCTA scan quality score of ≥7 at any visit; or 4) were lost to follow-up.

Patients were evaluated by slit lamp biomicroscope before surgery for cataract grading in Lens Opacities Classification System III [8]. Patients were also evaluated by indirect ophthalmoscope via dilated pupil at POD1 for diabetic retinopathy grading based on the Early Treatment Diabetic Retinopathy Study classification system [9]. Time from diagnosis of diabetes, glycemic control measured as hemoglobin A1c (HbA1c) concentration, and insulin dependence were recorded. HbA1c was tested with commercially available kit (Bio-Rad Laboratories, Inc., Hercules, CA).

### Surgical technique

All surgeries were performed by one surgeon (Z.W.) using Centurion Vision System (Alcon Laboratories, Inc., Texas, USA) under topical anesthesia. Briefly, after a 2.4 mm clear corneal incision was made, continuous capsulorhexis, hydrodissection, intracapsular phacoemulsification cataract extraction, irrigation/aspiration cortex removal, and posterior capsule polish were sequentially performed. Foldable hydrophilic acrylic intraocular lens (Akreos MI60, Bausch & Lomb, Rochester, N.Y., USA) was implanted in the capsular bag. Cumulative dissipated energy (CDE) and ultrasound total time (US) were recorded. Patients were administered topical diclofenac and levoflaxacin for 3 days before surgery and 4 weeks after surgery. Loteprednol etabonate eye drops were also applied and tapered for 4 weeks after surgery.

### Optical coherence tomography angiography image acquisition and vessel density measurement

Optical coherence tomography angiography scans were performed on the commercially available RTVue XR Avanti spectral domain OCTA device (Optovue Inc. Fremont, CA, USA). This device uses light source of 840 nm wavelength and operates at 70000 A-scans per second to generate OCTA volume scans composed of 304 × 304 A-scans over 3.0 × 3.0 mm macular area. At each visit, three consecutive macular scans centered on fovea with scan quality score ≥ 7 were obtained, and the one with lowest score was discarded. All image acquisitions were made by one single investigator (E.W.).

The quantitative measurements of vessel density were made by a built-in software AngioVue AngioAnalytics (Version 2017.1, Optovue Inc. Fremont, CA, USA) which uses split-spectrum amplitude decorrelation angiography algorithm with three-dimensional projection artifact removal technique. By default settings, foveal region is defined as the inner round area with 1.0 mm diameter, and parafoveal region is defined as an annulus area with inner and outer diameter of 1.0 mm and 3.0 mm, respectively. Superficial capillary plexus (SCP) extended from internal limiting membrane to 10 μm above inner plexiform layer. Deep capillary plexus (DCP) extended from 10 μm above inner plexiform layer to 10 μm below outer plexiform layer. The boundaries of capillary plexus were automatically recognized by the software and independently reviewed by two authors (E.W. and Y.C.) in case that manual adjustment was needed. The software automatically generated parafoveal SCP-VD and DCP-VD. Macular thickness of foveal and parafoveal area was also provided based on the same OCTA volume scan dataset. The measurements from two scans were averaged for further analysis. We did not include preoperative OCTA measurements because cataract could result in low signal strength and inaccurate quantification of OCTA metrics [10, 11]. The measurements at POD1 were used as baseline value for calculating VD changes and macular thickening.

### Statistical analysis

Statistical analyses were performed with SPSS software version 19 (SPSS, Inc., IL, Chicago, USA). Variable normality was inspected using Kolmogorov-Smirnov test. Baseline clinical characteristics were compared using one-way ANOVA and chi-square test for continuous variables and dichotomous variables, respectively. Postoperative VD changes in diabetic patients were evaluated using repeated measures ANOVA and Bonferroni’s post hoc test. The VD changes from baseline was compared between diabetic patients and controls using ANCOVA after adjusting for CDE and changes in scan quality score. Univariate Pearson correlation or Spearman correlation, where appropriate, was used to assess SCP/DCP-VD changes for their association with macular thickening and baseline clinical characteristics including age, gender, axial length, HbA1c level, duration since diagnosis of diabetes, insulin dependence, presence of NPDR at baseline, nuclear opalescence (NO); nuclear color (NC), CDE, and US. We then picked out candidate baseline characteristics for further multivariate linear regression to define the factors that significantly correlated with vessel density changes. A *P* value of less than 0.05 was considered as statistically significant.

## Results

The study enrolled 24 patients with type 2 diabetes, excluded 3 patients (1 lost to follow-up, 1 had OCTA scan quality score of lower than 7 at POD1 due to mild corneal edema, and 1 developed mild vitreous hemorrhage and received intravitreal injection at 6 weeks after cataract surgery due to rapid progression of diabetic retinopathy), and included 21 eyes of 21 diabetic patients for further analysis. The control group included 21 matched eyes of 21 non-diabetic subjects. At baseline, 5 of the 21 diabetic patients had mild or moderate NPDR, and the other 16 had no apparent retinopathy. At POD90, 1 of the 5 patients with NPDR at baseline developed mild cystoid macular edema at POD90. The baseline demographic characteristics and OCTA scan quality score at all follow-up visits are comparable between diabetic and control groups (Table 1).

**Table 1:** Demographics and OCTA scan quality score of diabetic and control Subjects

Characteristics	Diabetics (*n* = 21)	Controls (*n* = 21)	*P* values
Age (years)	70.0 ± 8.3	70.6 ± 6.0	0.80^*^
Gender (M:F)	11:10	8:13	0.35^†^
Axial Length (mm)	23.32 ± 1.30	23.72 ± 0.93	0.26^*^
Duration of diabetes (years)	15.1 ± 8.9	NA	NA
HbA1c (%)	7.31 ± 1.00	NA	NA
Insulin dependence	11 (52.4%)	NA	NA
NO	2.86 ± 0.69	2.79 ± 0.57	0.70^*^
NC	2.97 ± 0.71	2.87 ± 0.59	0.64^*^
CDE (second)	5.16 ± 1.59	4.65 ± 1.53	0.30^*^
US (second)	56.3 ± 11.8	51.7 ± 10.9	0.19^*^
OCTA scan quality score			
POD1	8.05 ± 0.55	8.12 ± 0.61	0.69^*^
POD10	8.10 ± 0.62	8.29 ± 0.60	0.32^*^
POD30	8.14 ± 0.74	8.14 ± 0.67	1.00^*^
POD90	8.14 ± 0.65	8.33 ± 0.56	0.32^*^

Data are shown as ratio, No. (%), or mean ± standard deviation

^*^P values are from one-way ANOVA

^†^*P* values are from Pearson Chi-square test

*NO* nuclear opalescence; *NC* nuclear color; *CDE* cumulative dissipated energy; *US* ultrasound total time; *NA* not applicable

### Macular vessel density changes

**Fig. 1:**
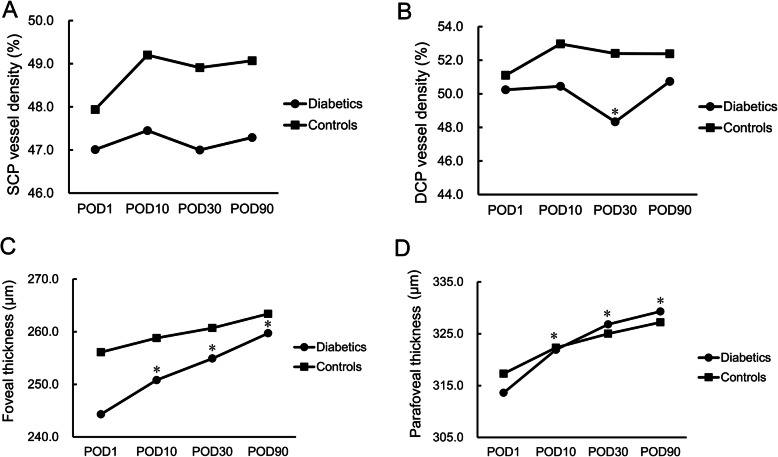
The longitudinal macular microvascular changes and macular thickness changes at POD1, POD10, POD30, and POD90 following phacoemulsification surgery in diabetic patients and non-diabetic controls. For the diabetic group, the significant changes compared with baseline are marked with asterisks. **a** vessel density changes in SCP; **b** vessel density changes in DCP; **c** foveal thickening; **d** parafoveal thickening. (POD = postoperative day; SCP = superficial capillary plexus; DCP = deep capillary plexus)

In patients with diabetes, no significant change in SCP-VD could be detected among the four postoperative visits (*P* = 0.57). A transient reduction in DCP-VD was noted at POD30 (*P* = 0.019) which restored to baseline level at POD90 (*P* = 1.00). A continuous increase in foveal and parafoveal thickness was also observed after surgery (both *P* < 0.001). The longitudinal changes of SCP-VD, DCP-VD, foveal thickness, and parafoveal thickness in diabetic patients and controls are shown in Fig. 1.

**Fig. 2:**
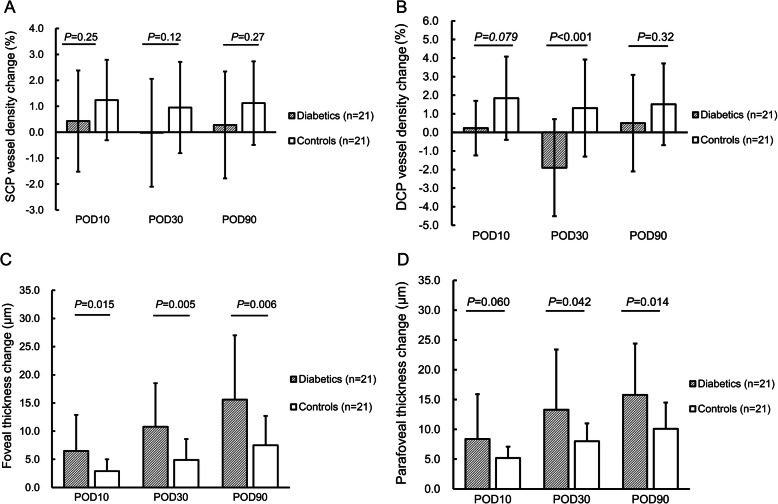
Comparison of macular microvascular changes and macular thickening at POD10, POD30, and POD90 following phacoemulsification between diabetic and non-diabetic patients. **a** vessel density changes in SCP; **b** vessel density changes in DCP; **c** foveal thickening; **d** parafoveal thickening. (POD = postoperative day; SCP = superficial capillary plexus; DCP = deep capillary plexus)

Comparison between diabetic patients and non-diabetic controls revealed no statistically significant difference in SCP-VD changes. However, the DCP-VD change at POD30 in diabetic patients (− 1.90 ± 2.61%) was significantly different from that in controls (1.31 ± 2.61%) after adjusting for CDE and changes in scan quality score (*P* < 0.001) (Fig. 2).

### Associated factors of macular vessel density changes

**Fig. 3:**
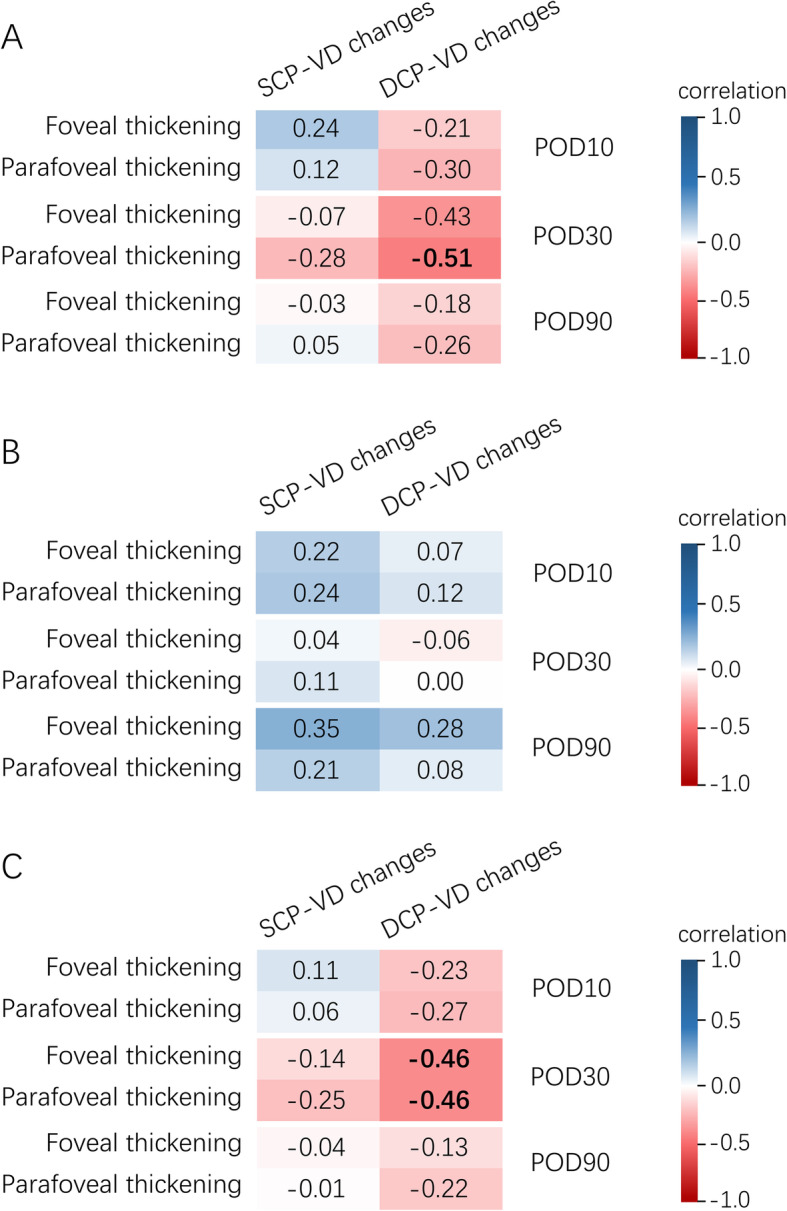
The correlation matrix plot for the association between macular thickening and vessel density changes in (**a**) diabetic patients (*n* = 21), **b** non-diabetic controls (*n* = 21), and (**c**) all study subjects (*n* = 42). Statistically significant correlations are shown in bold. (POD = postoperative day; SCP = superficial capillary plexus; DCP = deep capillary plexus; VD = vessel density)

In the diabetic group, univariate analysis indicated a correlation between DCP-VD reduction and foveal and parafoveal thickening at POD30 (*r* = 0.431, *P* = 0.051, and *r* = 0.514, *P* = 0.017, respectively). No significant correlation was found between SCP-VD changes and macular thickening (all *P* > 0.05). In the control group, univariate analysis detected no significant correlation between vessel density changes and macular thickening (all *P* > 0.05). Correlations between vessel density changes and macular thickness changes are shown in Fig. 3.

**Fig. 4:**
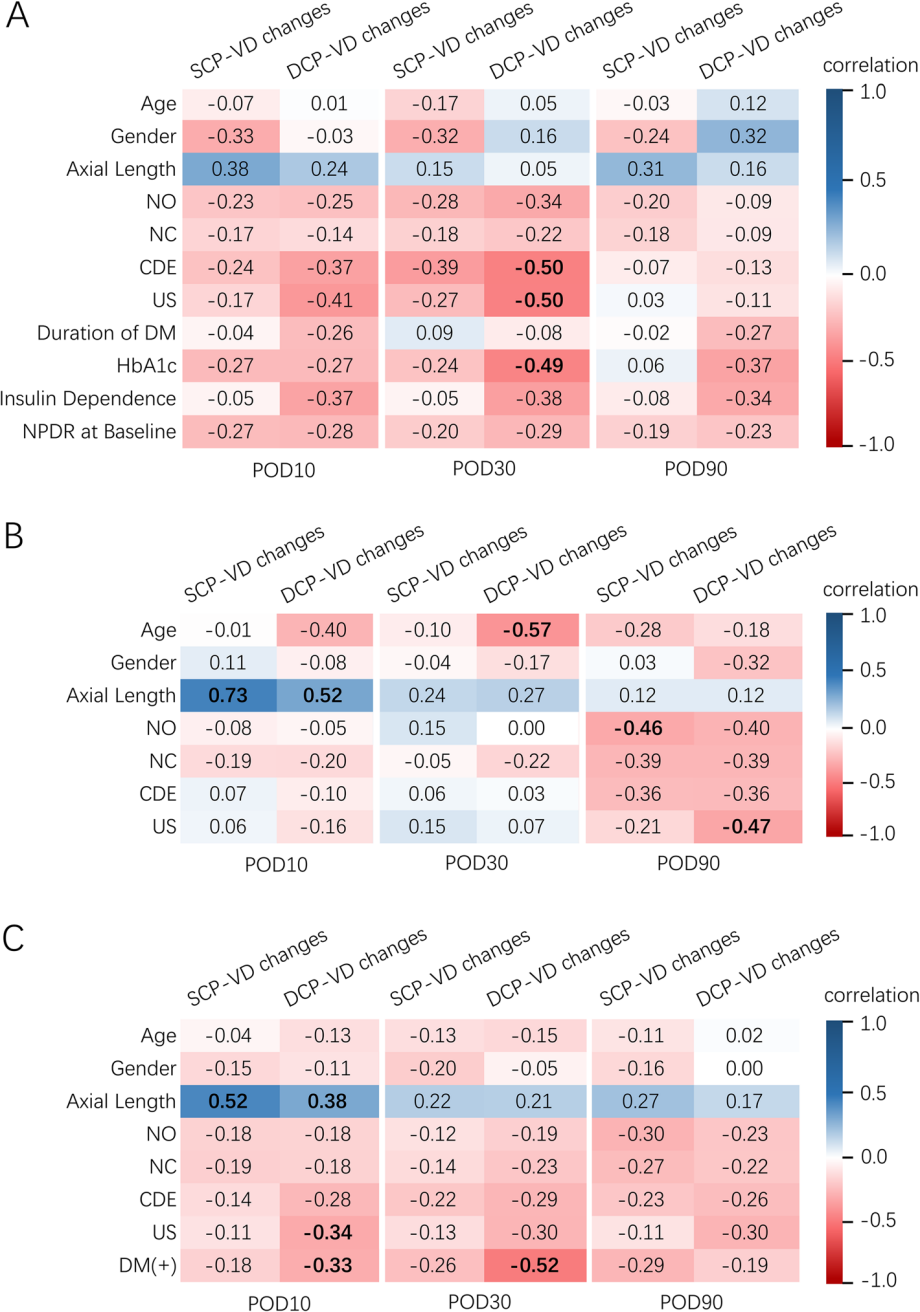
The correlation matrix plot for the association between vessel density changes and baseline clinical characteristics in (**a**) diabetic patients (*n* = 21), **b** non-diabetic controls (*n* = 21), and **c** all study subjects (*n* = 42). Statistically significant correlations are shown in bold. (POD = postoperative day; SCP = superficial capillary plexus; DCP = deep capillary plexus; VD = vessel density; NO = nuclear opalescence; NC = nuclear color; CDE = cumulative dissipated energy; US = ultrasound total time; DM = diabetes mellitus; HbA1c = Hemoglobin A1c; NPDR = nonproliferative diabetic retinopathy)

In the diabetic group, univariate analysis indicated that DCP-VD reduction at POD30 was significantly correlated with increased CDE (*r* = 0.503, *P* = 0.020), US (*r* = 0.500, *P* = 0.021) and HbA1c concentration (*r* = 0.492, *P* = 0.023). Other factors were not significantly correlated with vessel density changes in DCP or SCP. Multivariate linear regression in the diabetic group revealed that for every increase in HbA1c of 1%, the amount of DCP-VD reduction at POD30 would be added by 1.08% (95% confidence interval 0.07 to 2.08%, *P* = 0.037). For every increase in CDE of 1 s, the amount of DCP-VD reduction at POD30 would be added by 0.70% (95% confidence interval 0.07 to 1.33%, *P* = 0.032). When analyzing all the diabetic and control subjects as a whole group, univariate analysis indicated that DCP-VD reduction at POD30 was significantly correlated with the presence of diabetes (*r* = 0.523, *P* < 0.001) and possibly correlated with increased CDE (*r* = 0.290, *P* = 0.062) and US (*r* = 0.295, *P* = 0.058). Multivariate linear regression in the whole group revealed that the presence of diabetes was the only independent factor influencing DCP-VD reduction at POD30 (*P* < 0.001). Neither CDE (*P* = 0.13) or US (*P* = 0.16) had significant influence on DCP-VD change using multivariate model in the whole group. Correlations between vessel density changes and baseline clinical characteristics are shown in Fig. 4.

## Discussion

To the best of our knowledge, the current study is the first one that uses OCTA in describing microvascular changes following uncomplicated phacoemulsification cataract surgery in diabetic patients. We revealed that phacoemulsification surgery caused a transient reduction in the DCP-VD, which could be restored to baseline level at 3 months after surgery. We also found that the degree of DCP-VD reduction was associated with postoperative macular thickening, glycemic control, and surgical trauma.

Our findings suggested that the microvasculature networks in diabetic eyes responded differently to phacoemulsification surgery, mainly in the deep capillary plexus, compared with non-diabetic eyes. Previous studies reported a significant increase in macular vessel density in non-diabetic patients [6, 7]. Pilotto et al. [6] reported a 3–5% increase in the intermediate and deep capillary plexus following cataract surgery. In the study by Zhao et al. [7], parafoveal vessel density increased by approximately 5% at 1 month after phacoemulsification. In this study, non-diabetic controls showed mild increase in both SCP-VD and DCP-VD, however, diabetic eyes failed to show any increase in SCP-VD and even demonstrate a 1.90 ± 2.61% decline in DCP-VD. As opposed to the subtle changes in SCP-VD, the transient reduction in DCP-VD was recognized as a key feature in the postoperative microvascular changes in diabetic eyes. One possible explanation for the preferential involvement of DCP-VD was that DCP might be more sensitive to injury and more vulnerable to surgical trauma. Histopathological studies revealed that diabetic microaneurysms mainly originated from deeper part of retinal capillary plexus [12]. A growing body of OCTA studies also supported the distinct value of monitoring early microvascular changes in DCP in diabetic patients. Chen et al. [13] indicated that the fractal dimension changes in deep retinal capillary layer could be an early indicator of microvasculature changes associated with diabetic retinopathy. The OCTA metrics in DCP was not only an indicator of diabetic retinopathy severity [14], but also a predictor of diabetic retinopathy progression [15]. Furthermore, the low skeleton density in DCP was correlated with poor vision in patients with diabetic macular edema [16]. In concordance with previous studies, our results suggested the value of OCTA metrics on DCP in assessing microvascular changes following phacoemulsification surgery in diabetic patients.

The distinct pattern of microvascular changes in diabetic patients brought us to consider whether there was an association between postoperative macular thickening and macular vessel density changes in diabetic patients, especially at the level of deep macular capillaries. Previous OCTA studies have shown that non-diabetic PCME eyes have impaired deep macular microvascular density [17, 18]. In the study by Chetrit et al. [18], PCME eyes had significantly lower vessel density in DCP (44.1 ± 7.4%) than in controls (54.2 ± 3.2%), which could be restored (51.3 ± 6.1%) after resolution of edema. In another study by Sacconi et al. [17], PCME eyes had reduced vessel density in DCP which did not recover after treatment. Quantitative OCTA studies in diabetic patients have also identified reduced DCP flow in diabetic macular edema [16, 19]. In this study, macular thickness changes were analyzed, and our results were in line with prior structural OCT studies that diabetic patients had more pronounced macular thickening after cataract surgery compared with non-diabetic controls [20]. Moreover, this study detected a significant correlation between macular thickening and DCP-VD reduction. Our study not only supported, but also added new longitudinal evidence to previous cross-sectional observations that macular microvascular changes are involved in the complex pathophysiological process of macular thickening following phacoemulsification surgery.

It is noteworthy that the degree of DCP-VD reduction was only 1.90 ± 2.61% after adjusting for covariates, not as large as previously reported in PCME subjects [18]. Several reasons accounted for the mild reduction in DCP-VD in our study. First, only one of the 21 study subjects developed PCME, and the other 20 subjects had subclinical macular thickening without apparent macular edema. Second, the CDE during cataract surgery was low and postoperative inflammatory reaction was mild. Third, patients received nonsteroidal anti-inflammatory eye drops in addition to steroid eye drops, which had been reported to be more effective than steroid only in preventing PCME [21] and might also play a role in influencing the pattern of macular microvascular changes in response to phacoemulsification surgery.

In correlation analysis, we found that the presence of diabetes is the key factor associated with postoperative DCP-VD reduction. In addition, surgical trauma and glycemic control are important add-on factors influencing DCP-VD changes in diabetic patients. There has been evidence that diabetes alone does not impair recovery from cataract surgery [22]. Our results suggested that the presence of diabetes is an independent factor associated with DCP-VD reduction after cataract surgery. Poor glycemic control is a well-recognized risk factor for developing PCME in diabetic patients [23, 24]. Consistent with previous reports, our results reinforced the importance of overall glycemic control in preventing surgical related macular changes. As opposed to previous studies which did not identify surgical trauma as important risk factors for developing PCME [24], this study detected a significant correlation between DCP-VD reduction and CDE in diabetic patients, probably due to the homogeneity of phacoemulsification surgeries performed by one single surgeon. Insulin dependence was likely to be associated with DCP-VD reduction at POD30 (*r* = 0.382, *P* = 0.087), but the association did not reach statistically significant, possibly due to the small sample size. Previous study indicated an increased risk of developing PCME in diabetic patients with pre-existing diabetic retinopathy [25]. In this study, the presence of baseline NPDR failed to show significant association with microvascular changes, probably because the number of subjects with baseline NPDR was quite limited. Interestingly, in the diabetic group, the DCP-VD reduction at POD10 was likely to be correlated with CDE (*r* = 0.374, *P* = 0.095) and US (*r* = 0.411, *P* = 0.064), but not with HbA1c concentration (*r* = 0.272, *P* = 0.23), suggesting that microvascular changes were likely to be affected by surgical trauma in the early postoperative period and then influenced by the combined effect of surgical trauma and glycemic control level in the later recovery stage.

The strengths of our study were the prospective study design, longitudinal follow-up, and a high-quality data acquisition. We acknowledge several limitations in our study. First, the number of included subjects was limited. We did not include enough patients with baseline NPDR. Our results could not be readily generalized to diabetic patients with pre-existing diabetic macular edema or proliferative diabetic retinopathy. Second, we only evaluated parafoveal vessel density with a 3 × 3 mm field of view. The changes in perifoveal vessel density and the peripheral retina remained unknown. Third, we did not analyze the skeleton density changes. Fourth, we did not perform correlation analysis with visual outcome because the microvascular changes were subtle and the best corrected visual acuity of almost all patients reached 20/20 at POD90.

## Conclusions

Diabetic patients undergoing phacoemulsification surgery exhibited a transient mild reduction in vessel density of DCP at 1 month after surgery, which was correlated with postoperative macular thickening. Poor glycemic control and increased surgical trauma were found to be associated with the degree of DCP-VD reduction. Our findings offer new insights into the complex pathophysiological process of phacoemulsification surgery in diabetic patients and support the value of OCTA metrics in the management of postoperative changes in individuals with diabetes.

## Data Availability

The datasets used and/or analysed during the current study are available from the corresponding author on reasonable request.

## Abbreviations

ANCOVA: Analysis of covariance
ANOVA: Analysis of variance
CDE: Cumulative dissipated energy
DCP: Deep capillary plexus
HbA1c: Hemoglobin A1c
OCTA: Optical coherence tomography angiography
NC: Nuclear color
NO: Nuclear opalescence
NPDR: Nonproliferative diabetic retinopathy
PCME: Pseudophakic cystoid macular edema
POD: Postoperative day
SCP: Superficial capillary plexus
US: Ultrasound total time
VD: Vessel density
